# Supplementary material for: Understanding the perspectives and needs of multiple stakeholders: Identifying key elements of a digital health intervention to protect against environmental hazards
Source: PLOS Digit Health. 2024 Jan 29;3(1):e0000444. doi: 10.1371/journal.pdig.0000444 (PMC10824450; doi:10.1371/journal.pdig.0000444)
Supplement: S1 Table — (DOCX) [file pdig.0000444.s001.docx]

**S1 Table. Interview schedule for healthcare professionals**

| **Primary question/prompt** | **Additional questions** | **Clarifying questions** |
| --- | --- | --- |
| ***1. Attitudes toward applications***  Please describe your knowledge of smartphone health applications. | What is your general attitude towards mobile health apps?  Have you ever recommended a patient use a mobile health app to support their care? | Can you please tell me about your experience?      Can you please expand on this?      Can you please explain?      Can you please give me an example? |
| ***2. Knowledge of AirRater***  Please tell me what you know about AirRater. | Have you heard of AirRater? [If not, give a quick tour of the app]  How/when did you hear about AirRater?  Have you personally downloaded/used AirRater?  Do you ever recall a patient discussing AirRater with you? |  |
| ***3. Perceived effectiveness of AirRater***  Please share your perspectives on how useful you believe AirRater could be for users. | Do you think AirRater could/can help users to manage symptoms or to change behaviour?  Do you feel that there are features that could be improved or added to enhance the utility of AirRater for users?  Is there any additional information that AirRater could provide to users or collect from users that would assist you in your role as a clinician? |  |
| ***4. Support for AirRater***  Please describe your usual approach for managing patients with asthma, hay fever or another similar respiratory condition. | What tools and strategies do you generally recommend to patients for managing respiratory conditions, such as asthma or allergic rhinitis?  Have you ever recommended or prescribed AirRater or another smartphone application to a patient? |  |
| ***5. The value of AirRater***  Please describe your perspective on the potential value of AirRater. | Do you think AirRater supports the achievement of better health outcomes for users?  Can you identify any barriers that might impact the uptake or reach of smartphone health applications, such as AirRater?  Can you identify any enablers that might facilitate the uptake or reach of AirRater?  Do you see any opportunities for AirRater to be embedded into practice? |  |
| **6. Is there anything that we have not discussed that you think might be relevant or of interest?** | | |
| **7. Can you suggest any other colleagues who you think I might benefit from speaking with?** | | |
