# Supplementary material for: Understanding the perspectives and needs of multiple stakeholders: Identifying key elements of a digital health intervention to protect against environmental hazards
Source: PLOS Digit Health. 2024 Jan 29;3(1):e0000444. doi: 10.1371/journal.pdig.0000444 (PMC10824450; doi:10.1371/journal.pdig.0000444)
Supplement: S2 Table — (DOCX) [file pdig.0000444.s002.docx]

**S2 Table. Focus group discussion guide for healthcare professionals**

| **Welcome and introductions**  Thank you for taking the time to be here today. I am a researcher based at the University of Tasmania. I will be facilitating today’s group discussion. I’d like to introduce my colleague who will be taking notes on responses and observations during today’s discussion. |
| --- |
| **Question 1**  I’d like to start the conversation by understanding what experiences, if any, you as healthcare professionals have had with smartphone health applications more generally.  Going around the group, can you please tell us about your knowledge of and attitudes toward smartphone health apps, whether have used any personally, and whether you have recommended any to patients. |
| **Question 2**  Next, I’d like to focus in on AirRater and discuss what experiences, if any, you as healthcare professionals have had specifically with AirRater. Going around the group, can you please tell us whether you know about AirRater, whether have used it personally, and whether you have recommended it to patients. |
| **Question 3**  Next, I’d like you to share your perspectives on how useful you believe AirRater could be for users. Are there features that could be improved or added to enhance the utility of AirRater for users, or for you as a healthcare professional? How about additional information? |
| **Question 4**  I’d like us to discuss the potential value of AirRater more broadly as a public health intervention. Can you identify any barriers or enablers that might impact the uptake or reach of smartphone health applications, such as AirRater? Do you see any opportunities for AirRater to be embedded into practice? |
| **Question 5**  Are there any final points or comments you would like to make about AirRater or smartphone health applications more generally? |
