# Supplementary material for: Understanding the perspectives and needs of multiple stakeholders: Identifying key elements of a digital health intervention to protect against environmental hazards
Source: PLOS Digit Health. 2024 Jan 29;3(1):e0000444. doi: 10.1371/journal.pdig.0000444 (PMC10824450; doi:10.1371/journal.pdig.0000444)
Supplement: S3 Table — (DOCX) [file pdig.0000444.s003.docx]

**S3 Table. Interview schedule for agency and peak body representatives**

| **Primary question/prompt** | **Additional questions** | **Clarifying questions** |
| --- | --- | --- |
| ***1. Attitudes toward applications***  Please describe your knowledge of smartphone health applications. | What is your general attitude towards mobile health apps? | Can you please tell me about your experience?      Can you please expand on this?      Can you please explain?      Can you please give me an example? |
| ***2. Knowledge of AirRater***  Please tell me what you know about AirRater. | Have you heard of AirRater? [if not, provide a quick tour of the app]  How/when did you hear about AirRater?  Have you personally downloaded/used AirRater? |  |
| ***3. Support for AirRater***  Please share your perspectives on AirRater’s potential role in public health protection. | Do you think AirRater supports the achievement of better health outcomes for users?  What factors influence your decision to support/fund a public health intervention?  What benefits could/does AirRater bring your organisation?  Does AirRater create any negative impacts for your organisation? |  |
| ***4. Value of AirRater***  Please describe your perspective on the potential value of AirRater. | Do you see any opportunities for AirRater to be embedded into practice or to influence policy development?  Can you identify any barriers that might impact the uptake or reach of AirRater?  Can you identify any enablers that might facilitate the uptake or reach of AirRater?  Is there any additional information that AirRater could provide users or collect from users that would be beneficial for your organisation? |  |
| **5. Is there anything that we have not discussed that you think might be relevant or of interest?** | | |
| **6. Can you think of any other colleagues who I might benefit from speaking with?** | | |
