# Supplementary material for: Understanding the perspectives and needs of multiple stakeholders: Identifying key elements of a digital health intervention to protect against environmental hazards
Source: PLOS Digit Health. 2024 Jan 29;3(1):e0000444. doi: 10.1371/journal.pdig.0000444 (PMC10824450; doi:10.1371/journal.pdig.0000444)
Supplement: Attachment — Submitted filename: PLoS Digital Health_Responses to reviewer comments.docx [file pdig.0000444.s004.docx]

## Responses to reviewer comments

| **Reviewer** | **Comments** | **Response** |
| --- | --- | --- |
| Section Editor | We have noticed that you have uploaded Supporting Information files, but you have not included a list of legends. Please add a full list of legends for your Supporting Information files after the references list. | Thank you for this request. We have inserted the list of tables included in the Supplementary Material following the reference list. |
|  | In the online submission form, you indicated that "Requests for access to de-identified data presented in this study can be made to the corresponding author". All PLOS journals now require all data underlying the findings described in their manuscript to be freely available to other researchers, either 1. In a public repository, 2. Within the manuscript itself, or 3. Uploaded as supplementary information. This policy applies to all data except where public deposition would breach compliance with the protocol approved by your research ethics board. If your data cannot be made publicly available for ethical or legal reasons (e.g., public availability would compromise patient privacy), please explain your reasons by return email and your exemption request will be escalated to the editor for approval. Your exemption request will be handled independently and will not hold up the peer review process, but will need to be resolved should your manuscript be accepted for publication. One of the Editorial team will then be in touch if there are any issues. | Thank you for raising this issue. Our ethics application (ID: H0015006, approved by the University of Tasmania Health and Human Research Ethics Committee) stated that we would store both identified and de-identified data on secure servers and that they would only be available to researchers listed as members of the project team. This decision was made to protect the privacy of participants given we anticipated a small sample size. This information was communicated to all participants prior to their informed consent to participate in the interviews and focus groups.  We would need to email all participants and submit an amendment to the Ethics Committee to seek permission to publish de-identified data. Assuming all parties consented, which is not guaranteed, the process would likely take 4-6 months given turnaround times. We do not consider this a feasible undertaking.  After liaising with the PLOS Digital Health editorial team we have therefore opted to make our data available on request via the University of Tasmania Research Data Portal: <https://rdp.utas.edu.au/metadata/b540bd4e-bd00-42ff-b779-a5b9402ddb98> |
|  | Please present the methods section after the introduction and before the result section. | Thank you for your recommendation. We followed the PLOS guidance to authors in formatting our manuscript but agree that the methods section logically sits between the introduction and results sections and have moved it as requested. |
|  | Please provide a table of themes, subthemes, categories, etc. to show the summary of results. In fact, Table 1 needs to be revised to be more informative. | Thank you for your recommendation to update Table 1 (now Table 2). We have updated to more closely align the summary provided with the results presented. |
|  | Please explain how the researchers examined the trustworthiness of the qualitative results. | Thank you for this request. Using Lincoln and Guba’s (1985) trustworthiness criteria for qualitative research, we provided an accurate description of participants; considered and discussed the transferability of our results to other contexts, including an articulation of the limitations of our findings; and ensured results were reviewed by two or more people. |
|  | Please follow the journal instructions for citation within the text. | Thank you. We believe we have followed journal instructions for citations within text, using “Vancouver” style, as outlined by ICMJE. Please advise us if there is another preference, i.e. superscript numbers. |
| Reviewer #1 | Can you explain the term Peak Body for non-Australian audiences? i.e., non-governmental organisations, representative body etc. | Thank you for alerting us to this. We have updated the manuscript to provide a definition of a peak body for the benefit of an international audience. |
|  | In the introduction, can you be more specific about the background research and the benefits demonstrated through the research into this space.  Make it clearer what the current evidence shows and the gap in existing information that provides the justification for your study. | Thank you for this recommendation. We have updated the introduction section to provide further detail and clarity on the knowledge gap that this study aims to address. |
|  | Make it clearer what the aims and objectives of your study are. | Thank you for this suggestion. We have added the study objectives into the introduction section of the manuscript. |
|  | Add more information regarding how the participants were selected. Was any inclusion / exclusion criteria of participants considered? | Thank you, we have added in further details on the selection and recruitment of participants, including eligibility criteria. |
|  | What methodology was used to agree the coding system? Was this informed by any previous research? | Thank you, we have updated the manuscript to clarify that a blended (inductive-deductive) framework approach was used. This is the same approach we took to thematically coding and analysing the data from users (published in a separate manuscript). |
|  | Can you present the main coding themes that formed the basis of your results? | Thank you for this suggestion. The main codes/themes are presented in Table 1 (now Table 2). Following feedback from yourself and other reviewers, we have revised Table 1 (now Table 2) to provide further detail. |
|  | Review and improve the approach used for citing - (e.g. 1, 4). Perhaps the editor could comment better on this. | Thank you for this feedback. We have queried with the editor given we followed the PLOS Digital Health submission guidelines. |
|  | Suggest moving methods before results so that the reader can better follow how the research was undertaken prior to reading the results. | Thank you for this feedback. We have made this change as requested by the editor. |
|  | Describe / define what is meant by ‘vulnerable populations’ | Thank you for this suggestion. We have reworded the sentence, removing reference to ‘vulnerable populations’ and replacing with ‘populations considered to be at higher risk of adverse health outcomes from exposure to environmental hazards’. |
|  | Can you better align the questions you present with the headings used in the results? | Thank you. We have revised our research questions presented in the introduction section so that they more closely align with results. |
|  | For consideration – the authors may want to consider more granularity in presenting the results, separating the results into the aspects of the app stakeholders felt were useful and to whom, how this can deliver patient value - and what aspects were seen as negatives or barriers? e.g. benefits might be: early warning of poor air quality, improving health literacy, improved clinical symptom management. | Thank you for this suggestion. We have revised Table 1 (now Table 2) based on feedback from you and other reviewers. |
|  | Did clinician skills come up as an issue and if so how can this be addressed? | Thank you for this question. Clinician skills were not raised as an issue in the interview or focus groups. |
|  | Add to the discussion: Any conflicting view from those identified in previous research? If not, explore possible reasons for any conflicting views? | Thank you for this suggestion. We have updated the discussion section to clarify that there is limited literature exploring the perspectives of stakeholders beyond clinicians in relation to digital health interventions. |
| Reviewer #2 | This paper appears to be a reasonably well written qualitative evaluation of the AirRater app, which appears to provide some interesting data and can be useful to the groups interviewed in the paper. While I don't have a lot of specific comments regarding the paper, I have some larger general ones which I think the authors should address with revisions. The sample size of 20, even though it is fragmented across a few groups, is also better than other papers I have seen lately for qualitative research. | We thank Reviewer 2 for taking the time to review our manuscript. The suggested changes will strengthen the quality of the paper. |
|  | What kind of clinicians/patients (ie. what illness) are intended to use the AirRater app? When I was going through the paper the exact audience for the app was unclear to me. | Thank you for this question. We have updated the first paragraph to provide an example of some of the respiratory and cardiovascular conditions that are likely to be exacerbated by environmental hazards. |
|  | Why no images of app in the draft, or explanation of its dashboards? (perhaps they were in supporting documentation?) | Thank you for question. We confirm that we included images of the application in the supporting documentation. |
|  | Its good you have a table summarizing your findings under results, I just wonder if there is a way to pack more information into something like that considering that you have a lot of interview text to discuss. | Thank you for this suggestion. Based on your and other reviewers’ feedback, we have revised Table 1 (now Table 2) to contain further detail. |
|  | You swap "AirRater" for "environmental health smartphone apps" in a few places, such as in the abstract. Are you talking about apps "like AirRater" or is AirRater such a good example that it well-represents all "environmental health smartphone apps"? If so, justify, because the paper is somewhat unfocused in terms of addressing your research questions; you tend to jump around from an evaluation of "AirRater" to generalisations about "environmental apps" and the paper could probably be tightened up overall if you spend some time on this. Another example would be at the start of your discussion. | Thank you for raising this. While our study evaluated AirRater as an exemplar of an environmental health smartphone app, we were eager to consider the broader implications from our study for other environmental health apps, noting that readers are more likely to benefit from understanding the broader implications of our findings as they relate to environmental health apps, as opposed to AirRater specifically. We have attempted to clarify this throughout the manuscript. |
|  | Introduction/Abstract - Line 79 (for instance) - Please define "peak bodies" for international readers, or change the term to something more generic. This appears to be an Australian term. | Thank you for alerting us to this. We have updated the manuscript to provide a definition of a peak body for the benefit of an international audience. |
|  | Introduction/Abstract - Line 105 - Further app details and a schematic representation are documented elsewhere (10). - Can you please elaborate on what this means? | Thank you. We have updated this sentence to clarify that further app details are documented in an earlier publication. |
|  | Discussion - Line 408 - I would change "confirm" to "shows" | Thank you, we have made this suggested change. |
|  | Discussion - Line 463 - A critical gap in the literature with respect to apps or air quality apps? | Thank you, confirming that we believe our study results address a critical gap in the literature with respect to apps broadly, and have updated the sentence to make this clearer. |
|  | Discussion - Line 478 - "rather" – spelling | Thank you, we have updated the spelling accordingly. |
|  | Discussion - Line 490 - "utility" – spelling | Thank you, we have updated the spelling accordingly. |
| Reviewer #3 | Upfront, the authors should state the rationale for selecting the stakeholders. To this end, state what was the selection criteria of the stakeholders. Why was it essential to choose these stakeholders for his app? It needs to be clarified why these stakeholders were selected. If you address this as a multiple-stakeholder study, why is there more theoretical framing on clinicians? It is unclear why the one stakeholder in terms of literature and findings was given more voice and scaffolding in the paper, even why you separated the results as a clinician, government agency, and peak body representative. Is the app of greater priority to one stakeholder than the other? | Thank you for this suggestion. We have moved the methods section to below the introduction section, which includes details on the selection criteria for the stakeholders. We have also updated the introduction section to explain the rationale for the stakeholders we approached (see response to the next comment below) and clarified that there is virtually no literature that we have been able to identify that investigates the perspectives of government agency and beak body representatives on digital health interventions. This paper is one of the first to explore the perspectives of these stakeholder groups, making the comparison with literature on clinicians easier to undertake, hence the discussion gives more voice to the literature on clinicians. For the rest of the paper, we have attempted to provide an equal focus on clinicians, government agency and peak body representatives. |
|  | This description of the needs identified should be indicated earlier in the paper. This paper needs to clarify the rationale for the interface of a multi-stakeholder analysis. | Thank you for this suggestion. We have updated the introduction section to explicitly identify the gap that this study sought to address: understanding the needs of different stakeholders, who may have different reasons for using and valuing an environmental health app. |
|  | Indicate the critical elements for each stakeholder and include what need this fulfils for each stakeholder. It needs to be evident from the results how this app identifies key aspects of critical intervention. Instead, the paper is clear concerning knowledge and attitude and the barriers when stakeholders engage with the app. Consider revising the abstract and title. | Thank you for this suggestion. We have developed a second table that clarifies functions/application for individual users, clinicians and government and non-government representatives (please see **Table 1** in the manuscript). |
|  | After reading the questions, there appears to be a mismatch between the title, results reported and the questions. To the authors, if you are asking questions about the knowledge and attitudes, how does this relate to meeting the needs of these stakeholders? I suggest revising the title and abstract, considering the congruence not present between the research question and results in the paper. | Thank you for raising this issue. We have updated the title to ‘Understanding the perspectives and needs…’ to address this mismatch and confirm that the abstract is appropriate based on the revised title. |
| Reviewer #4 | This is a small, limited but still useful investigation of multi-stakeholder responses to a smartphone app that combines air monitoring with personal health data. It both offers behavioral guidance for users and collects data for public health surveillance and research. Developing tools for these functions is an important direction for environmental health and public health. Identifying barriers to adoption by clinicians is particularly important since they are gatekeepers and trusted communicators. The interest by government officials is encouraging. | Thank you to Reviewer #4 for taking the time to review and provide positive feedback on our manuscript. |
